# Effect of Red Blood Cells on Platelet Activation and Thrombus Formation in Tortuous Arterioles

**DOI:** 10.3389/fbioe.2013.00018

**Published:** 2013-12-03

**Authors:** Jennifer K. W. Chesnutt, Hai-Chao Han

**Affiliations:** ^1^Cardiovascular Biomechanics Laboratory, Department of Mechanical Engineering, The University of Texas at San Antonio, San Antonio, TX, USA; ^2^Department of Pathology, University of Texas Health Science Center at San Antonio, San Antonio, TX, USA; ^3^Biomedical Engineering Program, UTSA-UTHSCSA, San Antonio, TX, USA

**Keywords:** erythrocytes, shear-induced platelet activation, tortuosity, thrombosis, microvessels, mean platelet volume, computational simulation, discrete element model

## Abstract

Thrombosis is a major contributor to cardiovascular disease, which can lead to myocardial infarction and stroke. Thrombosis may form in tortuous microvessels, which are often seen throughout the human body, but the microscale mechanisms and processes are not well understood. In straight vessels, the presence of red blood cells (RBCs) is known to push platelets toward walls, which may affect platelet aggregation and thrombus formation. However in tortuous vessels, the effects of RBC interactions with platelets in thrombosis are largely unknown. Accordingly, the objective of this work was to determine the physical effects of RBCs, platelet size, and vessel tortuosity on platelet activation and thrombus formation in tortuous arterioles. A discrete element computational model was used to simulate the transport, collision, adhesion, aggregation, and shear-induced platelet activation of hundreds of individual platelets and RBCs in thrombus formation in tortuous arterioles. Results showed that high shear stress near the inner sides of curved arteriole walls activated platelets to initiate thrombosis. RBCs initially promoted platelet activation, but then collisions of RBCs with mural thrombi reduced the amount of mural thrombus and the size of emboli. In the absence of RBCs, mural thrombus mass was smaller in a highly tortuous arteriole compared to a less tortuous arteriole. In the presence of RBCs however, mural thrombus mass was larger in the highly tortuous arteriole compared to the less tortuous arteriole. As well, smaller platelet size yielded less mural thrombus mass and smaller emboli, either with or without RBCs. This study shed light on microscopic interactions of RBCs and platelets in tortuous microvessels, which have implications in various pathologies associated with thrombosis and bleeding.

## Introduction

Tortuous microvessels are found throughout the human body, such as coronary vasculature (Hutchins et al., 1978), and cerebral (Spangler et al., 1994; Brown et al., 2002), retinal (Sasongko et al., 2012), and conjunctival (Owen et al., 2008) arteries. Microvascular tortuosity alters blood flow to increase fluid shear stress that can activate platelets and induce thrombosis, even in the absence of vessel injury and hypercoagulability. For example, an *in vivo* study showed that thrombus formed due to high shear stress in rat venules that were made curved from an originally straight shape, but did not form in straight venules (Liu et al., 2008). As well, microvascular thrombi have been observed in humans in clinical, experimental, and autopsy settings (Gando, 2010). Consequently, the study of platelet activation and thrombus formation in tortuous microvessels is of clinical importance.

In straight vessels *in vivo*, large numbers of red blood cells (RBCs) flow in the vessel pushing platelets to the wall, known as platelet margination as demonstrated *in vitro* (Aarts et al., 1988). Thus, platelets are highly concentrated near the lumen walls. Responses of platelet margination and platelet adhesion rates to RBC collisions, hematocrit, and platelet size have been analyzed using mathematical models (Tokarev et al., 2011a,b). Also, the microscale processes of RBC and platelet interactions have been analyzed using computational simulations tracking individual cells. These simulations have been used effectively to examine the effects of RBC collisions, RBC aggregation, RBC deformability, RBC cytoplasm viscosity, platelet size and shape, platelet adhesion forces, and channel size on platelet margination, shear forces on platelets, and the process of thrombus formation (Miyazaki and Yamaguchi, 2003; AlMomani et al., 2008; Mori et al., 2008a; Chesnutt and Marshall, 2009a; Kamada et al., 2012; Reasor et al., 2012). Though these previous models have elucidated effects of RBCs in straight vessels and channels, effects of RBCs in tortuous vessels have not been addressed. In non-cylindrical vessels, a few *in vitro* studies with blood flow through sudden expansions showed that hematocrit affected spatial concentrations of RBCs and platelet-sized particles (Zhao et al., 2008) and affected platelet adhesion (Karino and Goldsmith, 1984). Hence, RBCs also likely play a role in thrombosis in tortuous vessels.

Most computational simulations that modeled thrombus formation by tracking individual platelets were performed in straight tubes or channels without RBCs (Miyazaki and Yamaguchi, 2003; Pivkin et al., 2006; Filipovic et al., 2008b; Fogelson and Guy, 2008; Mori et al., 2008b; Kamada et al., 2010), and very few included RBCs (Mori et al., 2008a; Xu et al., 2009; Kamada et al., 2012). Few simulations have been accomplished in non-cylindrical geometries (e.g., stenosis or tubular expansion), and these studies were without RBCs (Filipovic et al., 2008a; Kamada et al., 2011). In most of these models, platelets were activated due to an injured segment of the vessel wall. The effects of platelet activation by high shear stress were not studied.

Therefore, we have previously simulated the microscale processes of thrombus formation in tortuous arterioles and venules based on shear-induced activation of individual platelets (Chesnutt and Han, 2011, 2013). However, these studies did not include RBCs in the simulations.

Another physical factor that is present in conditions associated with many thrombotic and bleeding complications is platelet size, which is measured clinically as mean platelet volume (MPV). For normal healthy human subjects, an increase in MPV was shown to increase platelet aggregation in platelet rich plasma (Karpatkin, 1978). Elevated MPV is observed in pathological conditions, including diabetes (Papanas et al., 2004), hypertrophic cardiomyopathy (Cambronero et al., 2009), acute myocardial infarction (Chu et al., 2010), restenosis following coronary angioplasty (Chu et al., 2010), pulmonary hypertension (Guvenc et al., 2012), and giant platelet disorders (Mhawech and Saleem, 2000). MPV also decreases under other pathological conditions, such as reactive systemic amyloid A amyloidosis (Erdem et al., 2012) and Wiskott–Aldrich syndrome (Ochs et al., 1980). Hence, it is also of interest to determine the effects of platelet size in the presence of RBCs.

Consequently, the objective of this work was to determine the physical effects of RBCs, MPV, and vessel tortuosity on shear-induced platelet activation and thrombus formation in tortuous arterioles. These results would help to develop new treatment strategies and diagnostic tests for the risk of cardiovascular disease.

## Materials and Methods

The transport, collision, activation, adhesion, and aggregation processes of hundreds of individual RBCs and platelets of different MPV were numerically simulated in tortuous arterioles. The computational simulation conditions are described first, followed by brief descriptions of the employed discrete element model and shear-induced platelet activation model.

### Computational simulation conditions

**Figure 1:**
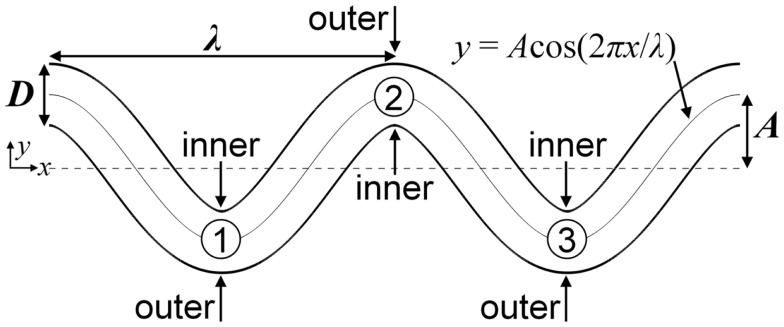
**Schematic of the high tortuosity arteriole showing the locations of inner walls, outer walls, and bends, which are labeled numerically**.

Segments of tortuous arterioles, each having diameter *D* = 25 μm, were modeled as two-dimensional (2D) channels in the shapes of cosine curves with two periods. Walls near the bends (locations with highest curvature) were designated as either inner or outer walls (Figure 1). A tortuosity index *T* was defined as the ratio of amplitude *A* to wavelength λ of the cosine curve (i.e., *T* = *A*/λ). To determine the effect of tortuosity, a low tortuosity arteriole with *T* = 0.09 and a high tortuosity arteriole with *T* = 0.21 were examined. Each arteriole had the same arc length. The assumption of 2D flow was used for computational efficiency and was expected to give qualitatively similar results and similar relative effects due to RBCs, tortuosity, and MPV compared with three-dimensional (3D) flow. The chosen arteriole diameter, tortuosity indices, and shapes were similar to those observed in tortuous arterioles in humans (e.g., Brown et al., 2002; Sasongko et al., 2012).

Platelets and RBCs were modeled as 3D spheres for computational efficiency, similar to previous computational studies of thrombus formation that utilized spheres or circles (Mori et al., 2008a; Kamada et al., 2010; Flamm et al., 2011). This approximation was expected to have a small effect on the results and is justified in more detail in the Section “Discussion.” In a given simulation, platelets had uniform properties, and RBCs had different uniform properties. To determine the effect of platelet size in pathological states, simulations used three different values of MPV found in humans (3.8, 7.1, 15.8 fl). The volume of an RBC was set to the physiological value in humans of 94 fl. The densities of platelets and RBCs are similar, and so were set to the same cell density ρ_c_ = 1063 kg m^−3^.

To determine the effects of both platelet distribution and presence of RBCs, three different seeding scenarios were employed to seed cells (i.e., platelets and RBCs) into the arteriole. In all seeding scenarios, cells entered the arteriole at the inlet with an initial velocity equal to the local fluid velocity at the location of the cell centroid, and seeded platelets were initially unactivated. In the whole-lumen-seeded platelets scenario, initial positions of platelet centroids at the inlet followed a pseudorandom probability distribution that excluded the region roughly one platelet radius from the wall such that platelets entered anywhere along the inlet boundary without touching the wall. RBCs were not included in the whole-lumen-seeded platelets scenario. In the near-wall-seeded platelets scenario, initial positions of platelet centroids at the inlet were bounded to regions within 7.5 μm of the walls. Centroid positions within these regions followed a pseudorandom probability distribution, without platelets touching the wall at their initial seed locations. This seeding assumed that the platelet distribution at the inlet was that expected in a straight arteriole with whole blood. RBCs, though, were not included in the near-wall-seeded platelets scenario. The RBCs with platelets scenario was the same as the near-wall-seeded platelets scenario, except that RBCs were also seeded into the flow. Positions of RBC centroids were initialized about the center of the arteriole, which spanned the 10-μm-region in which platelets were not seeded. Centroid positions of RBCs within this region followed a pseudorandom probability distribution. In each seeding scenario after a cell was seeded at the entrance, the cell was subjected to no other restrictions on its location or motion.

The number of unactivated platelets that entered the arteriole per unit time (seeding rate) was set such that a physiological time-averaged platelet count (300,000 mm^−3^) would be achieved in the absence of platelet adhesion to the walls. Hence, the seeding rate for whole-lumen-seeded platelets was required to be about twice that for near-wall-seeded platelets and for RBCs with platelets. The physiological time-averaged hematocrit was set to 6%. This hematocrit is representative of that in microvessels, which can be much lower than the ∼40% hematocrit of arteries and veins (Keller et al., 1994; Lipowsky, 2005).

Simulations were performed for Newtonian, incompressible, steady, fully developed, 2D flow in the *x*-*y* plane. Steady flow was assumed because flow pulsatility is minimal in the microcirculation. Due to low values of hematocrit and volume fraction of platelets, the effects of cells, aggregates, and mural thrombi on the fluid flow were neglected. This assumption is reasonable since the focus of this work was the initial formation of thrombus, rather than later possible occlusion. Centroids of 3D spherical cells remained the *x*-*y* plane in the 2D flow because forces on cells in the *z*-direction were zero, and the only non-zero torques on cells were in the *z*-direction. A Poiseuille velocity profile with typical mean arteriole velocity *U* = 0.6 cm s^−1^ was imposed on the fluid at the inlet of the arteriole. In a straight arteriole, this velocity profile would yield a wall shear rate |γ̇_0_| = 1440 s^−1^, which is in the range of typical values in arterioles (Kroll et al., 1996). Simulations considered blood to be a suspension of RBCs and platelets in plasma. Considered as a single-phase fluid, whole blood is a non-Newtonian fluid with variable apparent viscosity that depends on shear rate, which is mainly due the presence and aggregation of RBCs (Skalak et al., 1981). Rather than treat blood as a continuous fluid, the computational model simulated both plasma and cells, and incorporated a model that accounted for increased drag force on a cell due to increased apparent viscosity from cell crowding in the vicinity of the cell (Di Felice, 1994). Because of these modeling assumptions and the fact that plasma is a Newtonian fluid (Chien et al., 1966), the fluid was chosen to be Newtonian plasma with fluid density ρ_f_ = 1030 kg m^−3^ and dynamic viscosity μ = 1.2 cP. The parameter values utilized gave a Reynolds number (Re = ρ_f_*UD*/μ) ∼0.1 and particle Stokes numbers (St = ρ_c_*d*^2^*U*/18μ*D*) on the order of 10^−5^ for platelets and 10^−4^ for RBCs, where *d* is diameter of either a platelet or RBC. Simulation parameters related to physical properties of arterioles, cells, and plasma, as described above, are listed in Table 1.

**Table 1:** **Simulation parameters related to physical properties of arterioles, cells, and plasma**.

Parameter	Value	Comments
*D*	25 μm	–
*T* (low)	0.09	Low tortuosity arteriole, *A* ≈ 16 μm, λ ≈ 177 μm
*T* (high)	0.21	High tortuosity arteriole, *A* ≈ 30 μm, λ ≈ 141 μm
MPV (small platelet)	3.8 fl	Observed in Wiskott–Aldrich syndrome, corresponding to 1.9 μm spherical diameter
MPV (normal platelet)	7.1 fl	Physiological value, corresponding to 2.4 μm spherical diameter
MPV (large platelet)	15.8 fl	Observed in diabetes, corresponding to 3.1 μm spherical diameter
Volume of an RBC	94 fl	Corresponding to 5.6 μm spherical diameter
Platelet count	300,000 mm^−3^	0.2% Platelet volume fraction
Platelet seeding rate (whole-lumen-seeded)	0.67 ms^−1^	–
Platelet seeding rate (near-wall-seeded)	0.34 ms^−1^	–
Hematocrit	6%	1.71 ms^−1^ RBC seeding rate
ρ_c_	1063 kg m^−3^	–
ρ_f_	1030 kg m^−3^	–
μ	1.2 cP	–
*U*	0.6 cm s^−1^	–
|γ̇_0_|	1440 s^−1^	–
γ̇_crit_	1500 s^−1^	–
Re	0.1	–
St (platelet)	*O* (10^−5^)	–
St (RBC)	*O* (10^−4^)	–

Measures of thrombosis based on platelet activation, adhesion, and aggregation were determined throughout the simulation time. An aggregate was defined as a group of two or more platelets in contact with each other. A platelet was considered to be part of a mural thrombus if the platelet was in contact with the wall or if it was part of an aggregate that was in contact with the wall. One measure of thrombosis was mural thrombus mass, which was the number of platelets in mural thrombi at a given time multiplied by the mass of a platelet. This measure represented platelets that were in the vessel at the specified time. Another measure was average embolus mass, which was the number of platelets in an aggregate that was not in contact with the wall, averaged over the simulation time and multiplied by the mass of a platelet. Activation count was defined as the cumulative number of activation events. This measure was a count of the number of platelets that had become activated since the start of the simulation. To compare seeding scenarios that had different platelet seeding rates, activation percent was defined as the percent of seeded platelets that had become activated since the start of the simulation.

Simulations were run to a final time (*t*_f_ = 1.67 s) at which mural thrombus mass reached a state of statistical equilibrium for cases with the RBCs with platelets scenario. Simulation cases are listed in Table 2. Each simulation was performed as a serial (non-parallel) program on a 3.0-GHz node having either four or eight processing cores, with 2 GB of RAM per core. Simulations were run simultaneously on these processing cores. CPU times ranged from 8 to 17 days per simulation.

**Table 2:** **Simulation cases**.

Case	Tortuosity	Platelet size	Seeding scenario
1	Low	Normal	Whole-lumen-seeded platelets
2	Low	Normal	Near-wall-seeded platelets
3	Low	Normal	RBCs with platelets
4	High	Normal	Whole-lumen-seeded platelets
5	High	Normal	Near-wall-seeded platelets
6	High	Normal	RBCs with platelets
7	High	Small	Whole-lumen-seeded platelets
8	High	Small	Near-wall-seeded platelets
9	High	Small	RBCs with platelets
10	High	Large	Whole-lumen-seeded platelets
11	High	Large	Near-wall-seeded platelets
12	High	Large	RBCs with platelets

### Discrete element computational model

The transport, collision, activation, adhesion, and aggregation of hundreds of individual platelets and RBCs were simulated in tortuous arterioles by a mesoscale, discrete element method for blood cells. Details of the computational model have been previously published (Chesnutt and Marshall, 2009a,b; Chesnutt and Han, 2011). A brief description of the model is provided as follows.

The computational model followed the motion of each spherical cell due to interactions with the fluid, vessel walls, and other cells. Activated platelets were subjected to adhesion with the wall and aggregation with each other. RBCs were subjected to aggregation with each other, though shear rates were high enough in this work to suggest disaggregation of RBC aggregates (Schmid-Schonbein et al., 1968). In the remainder of this subsection, the term adhesion is used to refer to both adhesion and aggregation.

Cells were modeled as 3D spheres, rather than 2D circles, so that they possessed mass and could be subjected to the dominant 3D forces and torques as defined below. Fluid flow was modeled in 2D to increase computational efficiency. Cell centroids were initialized on the 2D flow *x*-*y* plane. As the fluid velocity component in the *z*-direction was zero, and the only non-zero fluid vorticity component was in the *z*-direction, centroids of 3D cells remained in the *x*-*y* plane. Thus, 3D angular and linear momentum equations were reduced to 2D and were solved for each 3D cell in 2D flow, given by

(1) $$m\frac{d\text{v}}{dt}={\text{F}}_{\text{F}}+{\text{F}}_{\text{A}},\;I_{z}\frac{d\Omega_{z}}{dt}=M_{\mathrm{F,}z}+M_{\mathrm{A,}z},$$

where *m* is cell mass, ***v*** is cell velocity, *I_z_* = (1/10)*md*^2^ is moment of inertia, Ω*_z_* is cell rotation rate, ***F***_F_ and *M*_F,*z*_ are respectively fluid-induced force and torque on the cell, ***F***_A_ and *M*_A,*z*_ are respectively force and torque on the cell due to adhesion and collision, and *t* is time. Fluid-induced force ***F***_F_ was composed of the dominant fluid-induced forces, which were drag force and added mass force. Adhesion and collision forces acting on two colliding cells consisted of a force due to elastic deformation of cells *F*_ne_, a force due to energy dissipation from collision *F*_nd_, and a resistance force *F*_s_ due to sliding of one cell over another. Adhesion and collision torques acting on two colliding cells consisted of a torque generated by the sliding resistance force *F*_s_ and a torque due to resistance of rolling of one cell over another *M*_r_. Adhesion and collision forces and torques were given by

(2) $$\begin{array}{l} {\text{F}}_{A}=F_{\mathrm{ne}}\text{n}+F_{\mathrm{nd}}\text{n}+F_{s}{\text{t}}_{s},\;M_{A,z}=\frac{d}{2}F_{s}\left(\text{n}\times {\text{t}}_{s}\right)+M_{r}\left({\text{t}}_{r}\times \text{n}\right), \end{array}$$

where ***n*** is a unit vector tangent to the line connecting the centroid of the cell to the centroid of the other cell, ***t***_s_ is a unit vector in the direction of relative motion of the cell surfaces projected onto the plane orthogonal to ***n***, and ***t***_r_ is a unit vector in the direction of rolling velocity of the cell. Forces (drag force, added mass force, *F*_nd_, *F*_s_) and torques (*M*_F,*z*_, *M*_r_) were determined using equations given in the literature. Elastic deformation force *F*_ne_ was determined, as described below, based on the theory of Johnson et al. (1971).

Upon collision, cells were assumed to retain their shapes except within a flattened circular region of contact, in accord with the model of cell–cell contact of Bell et al. (1984). Receptor-ligand bonds that formed within the contact region were modeled as springs following (Bell et al., 1984). Under our model assumptions, receptor-ligand binding took a form that was mathematically analogous to van der Waals adhesion with a time-dependent adhesive surface energy density α(*t*) given by

(3) $$\alpha \left(t\right)=-\frac{\sigma }{2}{\left(x_{b}-x_{e}\right)}^{2}\int_{0}^{1}N_{b}\left(t-t_{0}\left(s\right)\right)\;sds,$$

where σ is spring constant, *x*_b_ is length of the bond, *x*_e_ is equilibrium length of the bond, *N*_b_ is number density of bonds, and *s* is radial position with the contact region divided by the radius of the contact region. The variation of *N*_b_ with time was given by a kinetics equation of Bell (1978) that accounted for forward and reverse reactions of bond formation given by

(4) $$\frac{dN_{b}}{dt}=k_{f}\;\left(N_{\mathrm{L0}}-N_{b}\right)\left(N_{\mathrm{R0}}-N_{b}\right)-k_{r}N_{b},$$

where *k*_f_ and *k*_r_ are respectively forward and reverse reaction rate coefficients, and *N*_L0_ and *N*_R0_ are respectively initial ligand and receptor densities. The reaction rate coefficients were given by Dembo et al. (1988)

(5) $$\begin{array}{llll} k_{f} & =k_{\mathrm{f0}}\exp\left[-\frac{\sigma_{\mathrm{ts}}{\left(x_{b}-x_{e}\right)}^{2}}{2k\overset{¯}{T}}\right],\; & & \;\\ k_{r} & =k_{\mathrm{r0}}\exp\left[\frac{\left(\sigma -\sigma_{\mathrm{ts}}\right){\left(x_{b}-x_{e}\right)}^{2}}{2k\overset{¯}{T}}\right], \end{array}$$

where *k*_f0_ and *k*_r0_ are respectively initial forward and reverse equilibrium reaction rates, σ_ts_ is transition state spring constant, *k* is the Boltzmann constant, and $\bar{T}$ is absolute temperature. Number density of bonds in Eq. 4 was obtained using the solution given by Chesnutt and Marshall (2009a). For simulations in the current work, the time scale for bond formation was much less than the time scale for elastic response of two colliding cells. Thus, number density of bonds was approximated by its equilibrium value *N*_b_(∞), such that adhesive surface energy density simplified to a constant value, given by

(6) $$\alpha \left(t\right)=-\frac{\sigma }{4}{\left(x_{b}-x_{e}\right)}^{2}N_{b}\left(\infty \right),$$

where calculations yielded α(*t*) = −4 × 10^−1^ dyn cm^−1^ for platelets and −1 × 10^−4^ dyn cm^−1^ for RBCs.

Because of the analogy with van der Waals adhesion, we used the theory of Johnson et al. (1971) for adhesive elastic particles to approximate elastic deformation forces acting between two colliding cells, given by

(7) $$\begin{array}{llll} \frac{F_{\mathrm{ne}}}{F_{c}} & =4{\left(\frac{a}{a_{0}}\right)}^{3}-4{\left(\frac{a}{a_{0}}\right)}^{3∕2},\; & & \;\\ \frac{\delta_{n}}{\delta_{c}} & =6^{1∕3\;}\left[2{\left(\frac{a}{a_{0}}\right)}^{2}-\frac{4}{3}{\left(\frac{a}{a_{0}}\right)}^{1∕2\;}\right], \end{array}$$

where *a*(*t*) is contact region radius, and δ_n_ is normal overlap distance of cell surfaces. Critical force *F*_c_ and critical overlap δ_c_ can be written in terms of contact region radius at equilibrium *a*_0_ as

(8) $$F_{c}=3\pi \alpha R,\;\delta_{c}=\frac{a_{0}^{2}}{2\left(6^{1∕3\;}\right)R},\;a_{0}={\left(\frac{9\pi \alpha R^{2}}{E}\right)}^{1∕3},$$

where *R* and *E* are respectively effective radius and effective elastic modulus of the two cells, with adhesive surface energy density α given by Eq. 6.

Mechanisms of platelet aggregation and adhesion differ depending on shear rate (Jackson et al., 2009). As maximum shear rates in the current work ranged from about 1800–3100 s^−1^, platelets were assumed to adhere to von Willebrand factor immobilized on the subendothelium through glycoproteins GPIb or GPIIb-IIIa, and to aggregate with each other through bridging of GPIb or GPIIb-IIIa by plasma von Willebrand factor (Ikeda et al., 1991; Kroll et al., 1996; Konstantopoulos et al., 1997; Shankaran et al., 2003). At low shear rates, RBCs aggregate with each other, while at high shear rates these aggregates break up. Though different theories of the mechanisms of RBC aggregation exist (e.g., Chien and Jan, 1973; Neu and Meiselman, 2002), models of receptor-ligand binding of RBCs used in computational studies have produced aggregation behavior similar to experimental observations (Bagchi et al., 2005; Chesnutt and Marshall, 2009a, 2010). Hence, in the current work, RBC aggregation was assumed to occur through receptor-ligand binding.

Typical ranges of values obtained from the literature for parameters in Eqs 4 and 5, which vary widely for different cell adhesion problems, are given in Table 3, along with values used in simulations. Few values of parameters in Table 3 are known specifically for platelet adhesion or aggregation. We selected the density of platelet receptors and other parameters to achieve an effective spring constant for adhesion and aggregation of a platelet that was of the same order of magnitude as that determined in a previous study. Specifically, the effective spring constant for a normal sized platelet at the equilibrium contact region radius, calculated as $\sigma \pi a_{0}^{2}$*N*_b_ (∞), was 34 Nm^−1^. This value compared well with the platelet effective spring constant 50 Nm^−1^, which was determined by Filipovic et al. (2008b) from computational simulations by comparison with experiments. Parameters for RBCs were chosen to obtain the constant adhesive surface energy density α = −1 × 10^−4^ dyn cm^−1^, which previously showed good agreement with *in vitro* aggregation experiments (Chesnutt and Marshall, 2009a).

**Table 3:** **Values of parameters related to receptor-ligand binding used in simulations and ranges of experimental values**.

Parameter (units)	Value used in simulations for platelets	Value used in simulations for RBCs	Range of experimental values
*k*_f0_ (cm^2^ s^−1^)	10^−7^	10^−7^	10^−12^ to 10^−7^^a^
*k*_r0_ (s^−1^)	10^−5^	10^−5^	10^−5^ to 10^b^
*N*_L0_, *N*_R0_ (cm^−2^)	1.115 × 10^12^	4.798 × 10^9^	10^9^ to 10^12^^c^
*x*_e_ (nm)	20	20	5–50^d^
*x*_b_ (nm)	29	29	0 to length at which bond breaks
σ (dyn cm^−1^)	1.98	2.2	0.01–10^e^
σ_ts_ (dyn cm^−1^)	−5	−5	−5 to 5^f^
$\bar{T}\left(K\right)$	310	310	–

^a^From Hammer and Apte (1992).

^b^From Bell (1978), Ward and Hammer (1993).

^c^From Dembo et al. (1988), Doggett et al. (2002), Reininger et al. (2006), Michelson (2007), Fogelson and Guy (2008).

^d^From Bell et al. (1984), Evans and Leung (1984), Dembo et al. (1988).

^e^From Dembo et al. (1988), Chtcheglova et al. (2004).

^f^From Dembo et al. (1988).

### Shear-induced platelet activation model

We used our model of shear-induced platelet activation as in Chesnutt and Han (2011), which was shown in simulations to initiate thrombosis at inner walls in agreement with previous *in vivo* experiments. This activation model ensured the assumption that a platelet cannot become activated by a physiological shear rate or shear stress, irrespective of the amount of time a platelet is subjected to physiological shear stress. As well, this activation model clearly indicated the point at which an individual platelet became activated. These two attributes of our activation model are not necessarily apparent in other existing models that also account for exposure time to shear stress.

Our model assumed that a platelet became activated if it experienced a shear rate above a critical shear rate (γ̇_crit_) defined as γ̇_crit_ = *f* |γ̇_0_|, where *f* is a scaling factor greater than unity. We assumed a critical shear stress of τ = 60 dyn cm^−2^ to determine critical shear rate. With approximate apparent viscosity of whole blood μ_b_ = 4 cP, the critical shear stress corresponded to a critical shear rate γ̇_crit_ = τ/μ_b_ = 1500 s^−1^. Hence, the scaling factor was chosen to be *f* = 1.042. The critical shear stress was chosen based on *in vitro* experiments with whole blood and platelet rich plasma, which showed that shear-induced platelet activation and aggregation occurred at shear stresses that ranged from 15 to 30 dyn cm^−2^ (Chow et al., 1992; Konstantopoulos et al., 1995, 1997; Shankaran et al., 2003). Although some of these experiments also showed large increases in platelet activation and aggregation as shear stresses surpassed 75–140 dyn cm^−2^, our critical shear stress was chosen to illustrate trends in the early stage of thrombus formation with respect to changes in shear stress due to tortuosity.

To account for the presence of chemical agonists released by activated platelets, the model assumed a platelet became activated if it contacted another activated platelet, similar to the model of Kamada et al. (2010). In our simulations, only activated platelets were subjected to adhesion and aggregation, and activation was considered irreversible.

## Results

### Effects of RBCs and tortuosity with platelets of normal size

**Figure 2:**
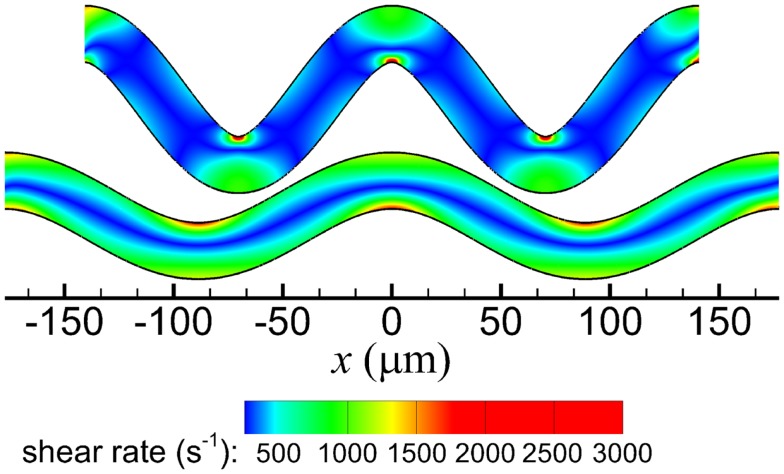
**Shear rate contours of the high tortuosity arteriole (top) and low tortuosity arteriole (bottom)**.

In both arterioles, maximum shear rates exceeded the wall shear rate that would occur in a straight arteriole (|γ̇_0_| = 1440 s^−1^) and the critical shear rate for activation (|γ̇_crit_| = 1500 s^−1^). Maximum shear rates were 1764 and 3099 s^−1^ in the low and high tortuosity arterioles, respectively. The highest shear rates occurred at inner walls in both arterioles (Figure 2), and fluid velocities in these regions were higher for the high tortuosity arteriole (not shown).

**Figure 3:**
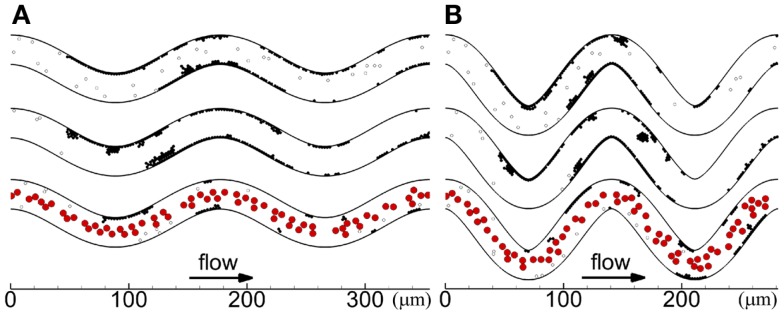
**Thrombus formation at *t*_f_ with different seeding scenarios, including whole-lumen-seeded platelets (top), near-wall-seeded platelets (middle), and RBCs with platelets (bottom), for low tortuosity index (A) and high tortuosity index (B), for Cases 1–6**. Cells include activated platelets (black), unactivated platelets (white), and RBCs (red). Platelets are normal size.

Cases with platelets of normal size for the two tortuosity indices and three seeding scenarios (Cases 1–6) are presented in this subsection. In each case, platelets first became activated at locations where shear rates were greater than the critical shear rate (critical shear regions) of the first and second bends. The first few mural thrombi formed on the inner walls of these two bends in some cases (Cases 2, 4, 6), and in other cases formed downstream of these two bends before reaching the third bend (Cases 1, 3, 5). After the onset of a thrombus at inner walls of the first or second bend, the thrombus first grew along the wall in the downstream direction and then later in the upstream direction. Throughout the simulations in each case, mural thrombi continued to form along the walls at other locations, as shown at the final time in Figure 3. For cases with RBCs, almost immediately after the first few mural thrombi formed, RBC collisions with the thrombi caused thrombi to roll along the wall. Throughout the simulations with RBCs, RBC collisions caused thrombi to continually roll along the wall and exit, generally in a single layer of platelets that could contain 10 or more platelets. For cases without RBCs, thrombi did not roll along the walls, except for one activated platelet and one three-platelet mural thrombus in Case 5.

For each case, emboli continually detached as mural thrombi grew into the flow (away from the wall), with some emboli reattaching on walls downstream. A single layer of platelets remained attached to the wall just after an embolus detached from a mural thrombus. For cases without RBCs, emboli consisted of an average of 8.4–8.9 platelets, with a maximum of 49 platelets. However, with RBCs, emboli were smaller and consisted of an average of 2.6 platelets, with a maximum of 6 platelets.

**Figure 4:**
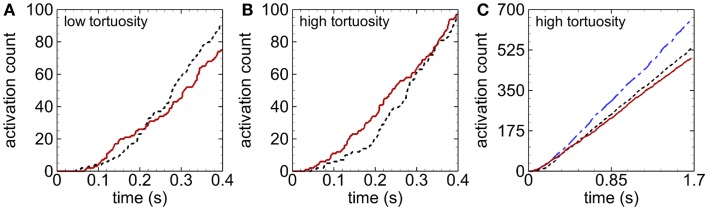
**Time variation of activation count during an early stage, for the low tortuosity (A) and high tortuosity (B) arterioles; and during the entire simulation time for the high tortuosity arteriole (C), for Cases 2–6**. Seeding scenarios include whole-lumen-seeded platelets (dashed-dotted line), near-wall-seeded platelets (dashed line), and RBCs with platelets (solid line). Platelets are normal size.

**Figure 5:**
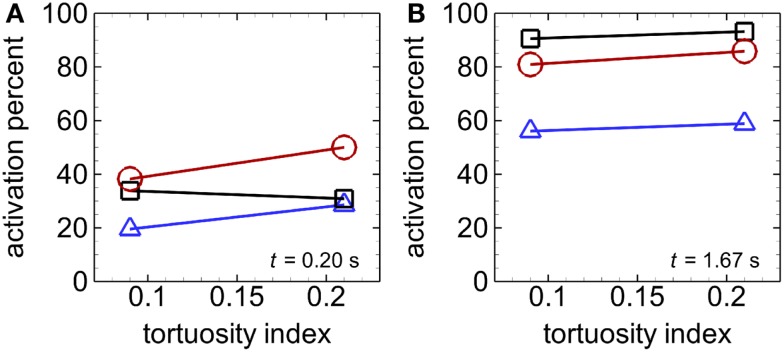
**Activation percent versus tortuosity index, with whole-lumen-seeded platelets (triangles), near-wall-seeded platelets (squares), and RBCs with platelets (circles), at an early time *t* = 0.2 s (A) and the final time *t*_f_ = 1.67 s (B), for Cases 1–6**. Platelets are normal size.

Activation count initially became larger for cases with RBCs compared with cases with only near-wall-seeded platelets, for a given tortuosity index (Figures 4A,B). This promotion of platelet activation due to RBCs lasted for a longer time in the high tortuosity arteriole (∼0.6 s) than in the low tortuosity arteriole (∼0.2 s). However, after this short time, activation count became smaller with RBCs compared to near-wall-seeded platelets without RBCs. Due to the higher seeding rate with whole-lumen-seeded platelets, activation count for this seeding scenario became larger than the near-wall-seeded platelets scenario and the RBCs with platelets scenario for both tortuous arterioles, as shown in Figure 4C for the high tortuosity arteriole. The low tortuosity arteriole showed similar results to those in Figure 4C, except that activation counts at the final time were slightly smaller at 623, 517, and 462, with whole-lumen-seeded platelets, near-wall-seeded platelets, and RBCs with platelets, respectively. These same values for the high tortuosity arteriole were 654, 532, and 490. However, activation percent was lowest at most times with whole-lumen-seeded platelets (Figure 5), due to the seeding of some platelets near the center of the arteriole.

**Figure 6:**
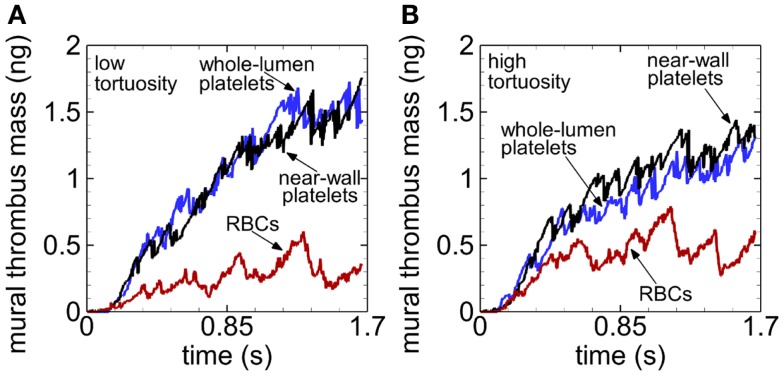
**Time variation of mural thrombus mass, with whole-lumen-seeded platelets (blue line), near-wall-seeded platelets (black line), and RBCs with platelets (red line), for the low tortuosity arteriole (A) and high tortuosity arteriole (B), for Cases 1–6**. Platelets are normal size.

For a given tortuosity index at any given time after 0.23 s at most, mural thrombus mass was smallest with RBCs compared to cases without RBCs, and about the same compared between cases without RBCs (Figure 6). Toward the end of the simulations, mural thrombus mass was generally larger for the low (versus high) tortuosity arteriole in the presence of RBCs, but generally smaller for the low tortuosity arteriole in the absence of RBCs.

### Effects of RBCs at different platelet sizes

**Figure 7:**
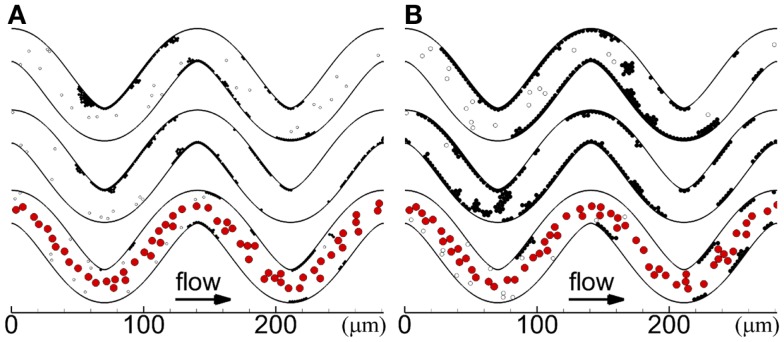
**Thrombus formation at *t* = 1.54 s for different seeding scenarios, including whole-lumen-seeded platelets (*top*), near-wall-seeded platelets (*middle*), and RBCs with platelets (*bottom*), for small platelets (A) and large platelets (B), for the high tortuosity arteriole for Cases 7–12**. Cells include activated platelets (*black*), unactivated platelets (*white*), and red blood cells (*red*).

Cases with platelets of different sizes (MPVs) for the three seeding scenarios in the high tortuosity arteriole (Cases 4–12) are presented in this subsection. In these cases, the first few mural thrombi formed either at the inner walls of the first and second bends or on walls downstream of bends after platelet activation at the first and second bends. The location of formation of the first few mural thrombi did not have a clear relationship with MPV or seeding scenario. Similar to the cases of normal MPV, RBC collisions with abnormal MPV caused mural thrombi to roll along walls and exit the arteriole. Additionally, without RBCs, most cases with abnormal MPV exhibited a small amount of rolling of thrombi, with the large MPV case exhibiting some rolling of large groups of platelets, though less than cases with RBCs. Locations of cells at the end of the simulations are shown for abnormal MPV in Figure 7.

**Figure 8:**
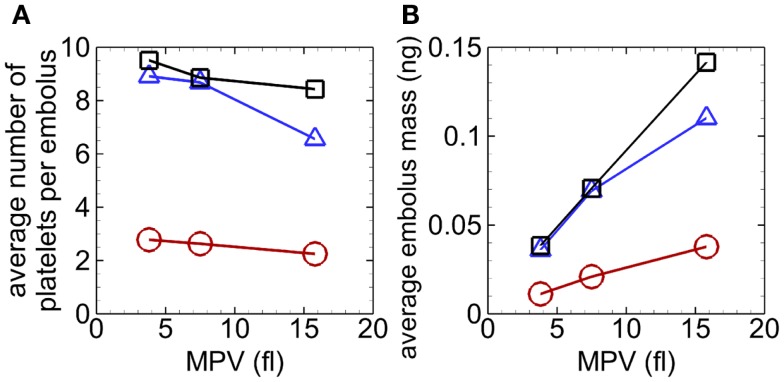
**Average number of platelets per embolus versus MPV (A) and average embolus mass versus MPV (B), with whole-lumen- seeded platelets (*triangles*), near-wall-seeded platelets (*squares*), and RBCs with platelets (*circles*), for the high tortuosity arteriole for Cases 4–12**. Values were averaged over the entire simulation time.

Emboli continually detached from mural thrombi for each case, with some emboli reattaching on walls downstream. Emboli in the absence of RBCs consisted of an average of 6.6–9.5 platelets (maximum of 37 platelets), and in the presence of RBCs an average of 2.3–2.8 platelets (maximum of 7 platelets) (Figure 8A). For a given seeding scenario, although the average number of platelets per embolus decreased with increasing MPV, average embolus mass increased with increasing MPV (Figure 8).

With near-wall-seeded platelets of large MPV, mural thrombi at the first bend eventually grew upstream along the wall and reached the inlet boundary at *t* = 1.54 s. In this case, locations of new near-wall-seeded platelets could not be seeded into the flow, and so simulations were analyzed at the time when thrombi first blocked the inlet near the wall, at *t* = 1.54 s, instead of the final simulation time *t* = 1.67 s. In future studies, the computational model can be modified to seed near-wall-seeded platelets toward the center of the arteriole in the event that locations near the walls are occupied by thrombi. Nevertheless, at time *t* = 1.54 s, mural thrombus mass had reached a state of statistical equilibrium for cases with the RBCs with platelets scenario so that results were expected to be valid. In cases without RBCs, mural thrombi at inner walls of the first and second bends grew in both the upstream and downstream directions. With RBCs, the small MPV case showed slight upstream growth of mural thrombi at the first and second bends before thrombi began to roll along the wall, while the large MPV case showed no upstream growth before thrombi began to roll along the wall.

**Figure 9:**
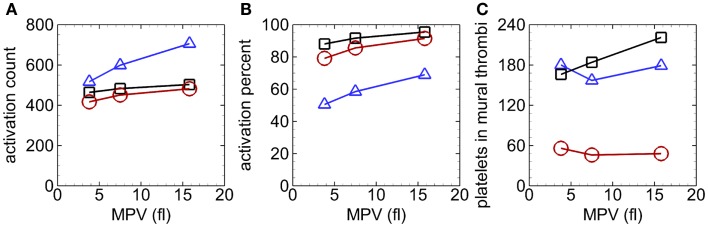
**Activation count versus MPV (A), activation percent versus MPV (B), and number of platelets in mural thrombi versus MPV (C), with whole-lumen-seeded platelets (*triangles*), near-wall-seeded platelets (*squares*), and RBCs with platelets (*circles*), in the high tortuosity arteriole at *t* = 1.54 s for Cases 4–12**.

Unlike the case with normal MPV near the beginning of the simulation, activation count with abnormal MPV was not clearly larger with RBCs compared to only near-wall-seeded platelets. As simulations progressed, activation count was largest for the case with whole-lumen-seeded platelets, followed by the case with near-wall-seeded platelets, and smallest for the case with RBCs, for a given MPV (e.g., Figure 9A). Activation percent at the end of the simulation was smallest for the case with whole-lumen-seeded platelets due to possible seeding in the center of the arteriole (Figure 9B). At the final time, activation count (and activation percent) increased as MPV increased for a given seeding scenario.

For a given MPV, mural thrombus mass increased with time with similar values for the two cases without RBCs and was smallest with RBCs. The trends with abnormal MPV (not shown) were similar to those with normal MPV (Figure 6B), except that values with small MPV were generally smaller and values with large MPV were generally larger, at a given time for a given seeding scenario. For example, values at the final time were at most 53% lower with small MPV (0.3–0.8 ng) and at most 176% higher with large MPV (1.0–3.6 ng). Though at the final time for a given seeding scenario, for cases with abnormal MPV compared with normal MPV, the number of platelets in mural thrombi did not vary as much as mural thrombus mass nor it have as clear a relationship with MPV (Figure 9C). Final numbers of platelets in mural thrombi with abnormal platelets varied from 23% lower to 25% higher than with normal platelets, depending on MPV and seeding scenario.

## Discussion

### RBCs, MPV, and tortuosity affect thrombus formation

Current results suggested that for normal sized platelets at the onset of thrombosis, RBCs collided with unactivated platelets to push them closer to walls where critical shear regions or mural thrombi were located. After a very short time, this effect was likely mitigated by formation of large enough mural thrombi that unactivated platelets could easily contact. Later, RBC collisions caused mural thrombi to roll out of the arteriole for all platelet sizes, which reduced platelet activation events, mural thrombus mass, and embolus sizes. Collisions of RBCs with mural thrombi caused emboli to detach before mural thrombi could grow as large as in the case without RBCs. However, without RBCs, mural thrombi were able to grow larger toward the vessel center where larger emboli were detached from mural thrombi by larger fluid forces, in combination with collisions with other platelets. The overall effect of RBCs on thrombus formation in tortuous arterioles may be to reduce the occurrence of thrombotic occlusion of the arteriole or of vessels downstream.

Results of the two seeding scenarios without RBCs indicated that thrombosis (and hemostasis in the case of injury) would proceed in a similar manner, but would be more efficient with near-wall-seeded platelets in a tortuous arteriole, as also expected in a straight vessel. Because the near-wall-seeded platelets scenario virtually excluded collisions of RBCs within the arteriole while it mimicked the effect of RBCs on the concentration profile of platelets at the inlet, a partial effect of RBCs would be to increase the efficiency of thrombosis and hemostasis in tortuous arterioles. However, because the RBCs with platelets scenario (i.e., near-wall-seeded platelets scenario with the effect of RBC collisions) caused less mural thrombus mass and smaller emboli sizes, our results suggested that RBCs would increase the efficiency of thrombus initiation, but reduce the risk of thrombotic occlusion due to arteriole tortuosity.

Our work suggested that, due to their small diameter, platelets of small MPV initially had a lower probability of collision with RBCs that could push them toward walls, and so RBCs did not increase activation count initially, as in the case of normal MPV. Moreover, RBCs did not increase activation count initially in the case of large MPV, which was possibly due to platelets of large mass undergoing smaller rebound velocities after collisions with RBCs that could push them toward walls. Our study showed that increased MPV yielded increased activation count at the final time for a given seeding scenario, including the two scenarios without RBCs and the scenario with RBCs. This result agreed with our previous study with whole-lumen-seeded platelets without RBCs (Chesnutt and Han, 2013), which was demonstrated at the tortuosity indices used in the current study, in addition to two intermediate tortuosity indices. Larger MPV may promote thrombosis through larger mural thrombus mass and larger emboli that would be more likely to occlude the arteriole or vessels downstream.

Our results revealed that tortuosity could generate high enough shear rates to induce platelet activation. Our results also suggested that, in the absence of RBCs, mural thrombi remained more intact in the arteriole of low tortuosity than in the arteriole of high tortuosity, likely due to forces at inner walls of bends (fluid and collision forces) that were lower in the arteriole of low tortuosity. Therefore, the fact that mural thrombus mass was smaller in the presence of RBCs in the arteriole of low tortuosity must be attributed to one or more of the following presumed events: more RBC collisions with mural thrombi, higher RBC collision forces with mural thrombi, or more rolling of mural thrombi out of the vessel at low tortuosity. Additional simulations to specifically measure individual collision events and collision forces or to virtually exclude specific events, such as thrombus rolling, may elucidate reasons for these results in future studies.

Our simulation of the process of thrombus formation yielded results that were similar to previous *in vivo*, *in vitro*, and computational studies. For example, thrombus formation was initiated at inner walls in some cases. This result agreed with a previous *in vivo* study in rats, which showed thrombosis was initiated at inner walls of curved venules due to high shear stress and high shear stress gradient in those regions (Liu et al., 2008). Another study with porcine whole blood perfused through glass stenoses that were coated with collagen showed that platelets initially adhered at the downstream end of the stenosis with some adhering upstream of the apex of the stenosis (Para et al., 2011). Later, thrombus formed both upstream and downstream of the stenosis apex. Although the very high shear rates in this previous study (∼100,000 s^−1^) indicated that platelet adhesion and aggregation occurred through different mechanisms than in our study (see Materials and Methods), the process of initial thrombus formation was similar to our study, in that thrombi that formed at the region of highest shear grew downstream first and then upstream later.

In a previous 2D computational study in simple shear flow with shear rate of 1000 s^−1^, platelets became activated by an injured wall segment to form a thrombus (Mori et al., 2008a). RBCs caused the thrombus to grow more along the wall than into the flow, and caused larger a thrombus mass. Our simulations agreed with the former result but not the latter. The discrepancy may be partially due to differences in the width of the region in which platelets were seeded, which was larger in the previous study (17 μm) than in our study (7.5 μm). Our smaller seeding width provided less space for thrombus to grow away from the wall and more opportunities for RBCs to collide with the layer of platelets adhered to the wall, causing thrombus to roll and exit the vessel. However, in the previous study of Mori et al. (2008a), RBCs were farther away from the thrombus base such that the thrombus was several platelet layers deep, and RBCs collided with platelets to push them toward the thrombus, rather than detach them from the wall.

A recent 3D computational study simulated individual platelets and deformable RBCs in straight cylindrical tubes of diameters between about 12 and 15 μm, with length 40 μm and Re about 0.01–0.02 (Kamada et al., 2012), which were smaller vessels and flow rates than in our study. In these previous simulations with and without RBCs, platelets became activated due to an injured wall segment, adhered to the injured wall, and initiated thrombus formation. The mural thrombus then grew downstream, with some platelets detaching from the thrombus due to collisions with RBCs or due to fluid forces. These initial processes of thrombus formation and effects of RBC collisions and fluid forces were in agreement with our results, though thrombosis was initiated by high shear stress in our study, rather than injury. However, the presence or absence of RBCs did not change thrombus height, area of injured wall covered by the thrombus, or number of platelets in the thrombus in this previous study. As noted by the authors, this result was due to the deformation of RBCs during collision with the thrombus. In contrast, our study assumed rigid RBCs and found smaller mural thrombus mass in the presence of RBCs. However, our Reynolds number (Re = 0.1) was 10 times higher than in the previous study, such that if deformable RBCs were used in our study, we would have observed larger RBC velocities that might produce large enough RBC collision forces to detach platelets from mural thrombi. Differences in results between the presence and absence of RBCs would be less pronounced with deformable RBCs than with rigid RBCs, but we would expect an effect of RBCs.

### Model limitations

The model of shear-induced platelet activation did not account for exposure time to shear stress, which has been shown to affect activation *in vitro* (Hellums, 1994). Our previous work (Chesnutt and Han, 2013) showed that our activation model was either in quantitative or qualitative agreement with a shear-induced platelet activation model that accounted for magnitude of and exposure time to shear stress (Alemu and Bluestein, 2007), depending on the parameters chosen for the model of Alemu and Bluestein.

The model contained various assumptions for computational efficiency. Platelets and RBCs were assumed to be spherical and rigid in the absence of collision or adhesion. Although unactivated platelets are discoid, activated platelets are more spheroidal (Michelson, 2007). Hence, the shapes of activated platelets are closer to spheres than disks. As the focus of our work was on thrombus formation by activated platelets, the difference in results due to use of a spherical platelet shape, rather than a spheroidal shape, were expected to be small. RBCs have a resting shape that is a biconcave disk, and can exhibit either a tumbling motion or tank-treading motion in fluid flow depending on shear rate (Bitbol, 1986). As well, RBCs are highly deformable, which is necessary for RBCs to traverse capillaries, which have smaller diameters than RBCs (Skalak et al., 1989). Because our study was simulated in arterioles with diameters larger than capillaries and focused on the effect of the overall presence of RBCs on thrombus formation, the assumption of spherical non-deformable RBCs was expected to have a small effect on the results.

Fluid flow was assumed to be 2D and unaffected by the presence of cells (*one-way coupled*). Under these assumptions, our previous work showed that aggregates of cells in 2D shear flows were quantitatively similar in size and shape to those in 3D flows (Chesnutt and Marshall, 2010), and that thrombosis was initiated at the same locations in 2D channel flows as *in vivo* (Chesnutt and Han, 2011). Also, the effects of cells on the fluid were reduced due to the low volume fractions of platelets and RBCs in our simulations. Because this work focused on initial thrombus formation, rather than occlusion, the assumptions of 2D flow and one-way coupling were expected to give qualitatively similar results and similar relative effects due to RBCs, MPV, and tortuosity compared to 3D flow and two-way coupling.

### Clinical relevance

The physical effects of RBCs, MPV, and tortuosity may compound the chemical factors and other physical factors present in disease states. In the absence of RBCs, our results showed much larger mural thrombus mass and embolus size, which suggested a higher risk of thrombotic complications. This result was in accord with a retrospective study of anemic patients who underwent free flap reconstructive surgery (Hill et al., 2012). Preoperative values of hematocrit and hemoglobin were significantly lower in patients who experienced thrombosis and who experienced flap failure, which was primarily due to thrombosis. The authors proposed that thrombosis could have been partly due to increased turbulence from lower blood viscosity or due to alterations in platelet aggregation and concentrations of coagulation factors. In addition to these factors, our results suggest that fewer RBC collisions due to lower hematocrit might promote thrombosis once thrombi begin to form during free flap procedures in anemic patients.

In cases with RBCs, mural thrombus mass and embolus mass were about double for cases with large platelets compared to cases with normal platelets. Hence, the physical effects of large platelets and microvascular tortuosity in diabetes may be partly responsible for the large incidence of thrombosis, which is also attributed to hypercoagulability in diabetes. Because metformin reduces platelet size in diabetes (Dolasik et al., 2013), this drug may also reduce the risk of thrombosis due to the physical effects of platelet size. This mechanism was speculated in our previous work that neglected RBCs (Chesnutt and Han, 2013) and is confirmed by the current work that includes RBCs. Mural thrombus mass was about half for cases with small platelets compared to cases with normal platelets, throughout the simulations with RBCs. Assuming a similar effect under conditions of platelet activation due to vessel injury, rather than high shear stress, this physical effect of small platelet size may contribute to the high risk of bleeding observed in Wiskott–Aldrich syndrome, which is also attributed to severely low platelet count and other factors (Ochs et al., 1980).

### Conclusion and outlook

The computational model simulated individual platelets and RBCs in the microscale processes of shear-mediated thrombus formation in tortuous arterioles. We concluded that RBCs initially promoted platelet activation, but that this effect was quickly reversed as simulations progressed. Collisions of RBCs with mural thrombi yielded reduced mural thrombus mass and embolus mass. As well, a reduction in platelet size resulted in reduced mural thrombus mass and embolus mass, either with or without RBCs. Hence, vessel occlusion may be less likely due to the presence of RBCs and due to smaller platelet size. A smaller mural thrombus mass was observed at the low tortuosity index compared to the high tortuosity index in the presence of RBCs. Our results suggest that the physical mechanisms associated with RBCs, platelet size, and vessel tortuosity play important roles in platelet activation and thrombus formation.

To our knowledge, except for our two previous works (Chesnutt and Han, 2011, 2013), no other computational studies have simulated shear-induced activation of individual platelets in thrombus formation, possibly because previous activation models did not clearly indicate the incident of activation. With the clear occurrence of platelet activation in our computational model, we were able to simulate a large number of platelets in thrombus formation. Our study shed light on mechanical factors involved in tortuosity-induced thrombosis, including the presence of RBCs, platelet size, and microvessel tortuosity. The model serves as a foundation to build upon in future work, for example with improvements such as more physiological shapes of RBCs and platelets and/or 3D fluid flows. Future studies may help determine the risk of thrombotic or bleeding complications due to physical mechanisms associated with RBCs, platelets, and vessel tortuosity, to aid treatment and prevention of these complications in various pathologies.

## Author Contributions

Hai-Chao Han and Jennifer K. W. Chesnutt contributed to study conception and design, interpretation of data, and drafting and critical revision of the manuscript. Jennifer K. W. Chesnutt acquired and analyzed data.

## Conflict of Interest Statement

The authors declare that the research was conducted in the absence of any commercial or financial relationships that could be construed as a potential conflict of interest.
